# Supplementary material for: Examination of factors that impact mask or respirator purchase and usage during the COVID-19 pandemic
Source: PLoS One. 2024 Sep 25;19(9):e0308841. doi: 10.1371/journal.pone.0308841 (PMC11423998; doi:10.1371/journal.pone.0308841)
Supplement: Attachment — Submitted filename: Response to Reviewers.docx [file pone.0308841.s001.docx]

Response to Reviewers

**Reviewer #1:**

**Methodology:**

1. As you already mentioned the exclusion criteria in lines 111-112 “Sample selection excluded individuals below the age of 18 and non-English speakers” mentioning it again as the inclusion criteria is not needed in line 116 “were only 116 English speakers and adults older than 18”

- **Response:** Exclusion criteria was rewritten in line 126-127 to reflect “The sample inclusion criteria was set as individuals above the age of 18 and English speakers.”

2. Please rewrite the Table 1 title as Variables Analyzed instead of “Dependent Variables Analyzed”

- **Response:** The title of Table 1 was rewritten as “Variables Analyzed” from “Dependent Variables Analyzed”.

3. In “Table 1: Dependent Variables Analyzed” what is the basis of categorizing the income of the respondent into two groups “Under $ 35,000 annually” and “Over $35,000 annually”

- **Response:** The dichotomizing of income into “Under $35,000 annually” and “Over 35,000” was identified based on the 2023 US income levels determined by a report from Hud User. The cut off of 35,000 reflects a rounded value between what was defined a “Very low income” and “Low Income” for average US annual income.
  - Line 160-164 “The independent variables were assessed using a combination of dichotomous, 5-point Likert scale, and multiple-choice questions. Income was dichotomized into “Under $35,000” and “Over $35,00” based on income limits reported by the U.S. Department of Housing and Urban Development’s Office of Policy Development and Research.^[28]”^
  - Linke 445-447 Citation was added FY 2023 State Income Limits . U.S. Department of Housing and Urban Development’s Office of Policy Development and Research. 2023. Accessed July 6, 2024. https://www.huduser.gov/portal/datasets/il/il23/State-Incomelimits-Report-FY23.pdf.

4. Please mention how the participants were recruited.

- **Response**: The following explanation of the participant recruitment was added:
  - **Line** 133-137 “A convenience sample was gathered through the Qualtrics Research Platform. Individuals were previously recruited to a survey panel and sent an email/link asking them to complete the survey. Survey participants were compensated for serving on survey panels by Qualtrics Research Platform. The participants did not receive any compensation by the authors in this study. “

5. Please mention if the survey questionnaire was pretested or validated prior to the data collection.

- **Response:** The explanation of pretesting was provided
  - Line 124-126 “An initial pretesting on a smaller population was conducted, which allowed the authors to identify possible wording bias in the survey questionnaire.”

6. Please clearly mention the study design at the start of the Methods section to help readers quickly understand the approach.

- **Response:** A statement identifying the study as a cross-sectional study design was added
  - Line 108-109 “The study was a cross-sectional study design, and consisted of a survey administered through the Qualtrics Research Platform.”

7. Kind describe shortly regarding the sample size was calculation. Although some information was mentioned in the Limitations sections, it would be best to include a concise description in the methodology as well.

- **Response:** An explanation of how the sample size was determined was added
  - Line 111-113 “The sample size was determined through a combination of survey sample best practices, financial considerations, and calculation of recommended sample size based on ±5% Margin of Error and the large study population size. ^[28]^”
  - Citation was added Line 443-444 “Story DA, Tait AR. Survey Research. *Anesthesiology*. 2019;130(2):192-202. doi:10.1097/ALN.0000000000002436”

**Discussion:**

1. In line 214 it is mentioned that “These results are interesting because females have been previously associated with a higher frequency of mask-wearing behavior” and the following paper was cited “McMahon E, Wada K, Dufresne A. Implementing fit testing for N95 filtering facepiece respirators: practical information from a large cohort of hospital workers. Am J Infect Control. 2008;36(4):298-300. doi:10.1016/j.ajic.2007.10.014”. However, this article does not contain any supporting findings. Please cite reference number **31** “Looi, K.H. Explicating gender disparity in wearing face masks during the COVID-19 pandemic. BMC Public Health 22, 2273 (2022). https://doi.org/10.1186/s12889-022-14630-7” which has relevant findings.

- **Response:** In line 233
- “McMahon E, Wada K, Dufresne A. Implementing fit testing for N95 filtering facepiece respirators: practical information from a large cohort of hospital workers. Am J Infect Control. 2008;36(4):298-300. doi:10.1016/j.ajic.2007.10.014” reference was removed and “Looi, K.H. Explicating gender disparity in wearing face masks during the COVID-19 pandemic. BMC Public Health 22, 2273 (2022). <https://doi.org/10.1186/s12889-022-14630-7>” was cited.

2. In line 224-226 it is mentioned that “Previous studies have associated headaches, itching, increased facial temperatures, and humidity with the 195 N95 and could be contributing to some choosing looser fitting masks over respirators” followed by in text citation of reference number 30 and 32. However, reference number 30 does not have any supporting findings. Please remove reference number 30 [“Christopher L, Rohr-Kirchgraber T, & Mark S.. The PPE pandemic: Sex-related discrepancies of N95 mask fit. European Medical Journal. https://www.emjreviews.com/microbiology-infectious-diseases/article/the-ppe 428 pandemic-sex-related-discrepancies-of-n95-mask-fit-j100121/. Published 2021 Dec. 22. Accessed 2023 Aug. 15”] and **keep only 32** [“Scheid JL, Lupien SP, Ford GS, West SL. Commentary: Physiological and psychological Impact of Face Mask Usage during the COVID-19 Pandemic. Int J Environ Res Public Health. 2020;17(18):6655. Published 2020 Sep 12. doi:10.3390/ijerph17186655”] here, or if needed add other relevant references.

- **Response:** In line 246 reference removed
- “Christopher L, Rohr-Kirchgraber T, & Mark S.. The PPE pandemic: Sex-related discrepancies of N95 mask fit. European Medical Journal. https://www.emjreviews.com/microbiology-infectious-diseases/article/the-ppe 428 pandemic-sex-related-discrepancies-of-n95-mask-fit-j100121/. Published 2021 Dec. 22. Accessed 2023 Aug. 15” reference was removed and “Scheid JL, Lupien SP, Ford GS, West SL. Commentary: Physiological and psychological Impact of Face Mask Usage during the COVID-19 Pandemic. Int J Environ Res Public Health. 2020;17(18):6655. Published 2020 Sep 12. doi:10.3390/ijerph17186655” was used instead.

Your manuscript demonstrates considerable promise, and I am confident that addressing these points will enhance the robustness of your research.

**Reviewer #2:**

The article is on impact of various motivational factors on individual choice of masks or respirators during the COVID-19 pandemic. The article is important to scientific community, however lacks many important details and analysis. It can be improved by heeding my comments, suggestion, and queries.

1) The 1st sentence of the introduction mentions the place from where the pandemic emerged. There is still lack of proof of source of COVID-19, and mentioning a certain city as source may not be appropriate. The source is considered to be from a non-human species which could be native to any country. Instead of direct reference, use words like 'may have', or 'most probably have emerged'

- **Response** The first sentence was changed to reflect the uncertainly in the viral origin.
  - Line 41-42 “In January 2020, COVID-19 disease, emerging most probably from in Wuhan, China, affected the world within months.”

2) The requirement and selection of masks and respirators cannot be generalised to USA only. different nations have been using masks on daily basis even pre COVID-19. The current study is only valid for the 619 English speaking participants and their country.

- **Response**: Although our current survey participants are mainly from the United States, we are aware of the limitation to the current data’s generalizability. We will note of this limitation in the discussion section**.**
  - Line 286-288 “The study used a convenience sample consisting of 619 participants, which limits the generalizability of the current study”.

3) Air flow resistance has less to do with masks then with respiratory disease. The term 'airflow resistance' should be removed. Stick only with 'breathability'.

- **Response:** The wording airflow resistance was removed
  - Line 74-75 “ In many individuals, comfort, such as the breathability and the amount of heat or moisture retained in the mask are cited as a key factors in a mask or respirator choice ^[16-20]^.”

4) The exclusion criteria is already mentioned in lines 111-112 “Sample selection excluded individuals below the age of 18 and non-English speakers” mentioning it again as the inclusion criteria is not needed in line 116 “were only 116 English speakers and adults older than 18”

- **Response:** Exclusion criteria was rewritten
  - line 126-127 “The sample inclusion criteria was set as individuals above the age of 18 and English speakers

5) There is no clarity on how the participants were recruited. please clarify this.

- **Response**: The following explanation of the participant recruitment was added:
  - Line 133-137 “A convenience sample was gathered through the Qualtrics Research Platform. Individuals were previously recruited to a survey panel and sent an email/link asking them to complete the survey. Survey participants were compensated for serving on survey panels by Qualtrics Research Platform. The participants did not receive any compensation by the authors in this study.”

6) Please give explanation on the validation of data collection.

- **Response:** The explanation of pretesting was provided
  - 124-126 “An initial pretesting on a smaller population was conducted, which allowed the authors to identify possible wording bias in the survey questionnaire.”

7) The methodology is not defined clearly, which creates confusion to go through the manuscript. also, mention the sample size to show the reliability of the study.

- **Response**: In this updated manuscript, we have mentioned a current survey study as a cross-sectional survey research. Additionally, we have added the details of research procedures, measures, and participants to clarify research design, measures, and sampling procedure using Qualtrics research panel platform.
