# Supplementary material for: Examination of factors that impact mask or respirator purchase and usage during the COVID-19 pandemic
Source: PLoS One. 2024 Sep 25;19(9):e0308841. doi: 10.1371/journal.pone.0308841 (PMC11423998; doi:10.1371/journal.pone.0308841)
Supplement: Attachment — Submitted filename: Response to Reviewers2.docx [file pone.0308841.s002.docx]

**Response to Reviewers**

**1. Additionally, please provide explanation of data validation.**

- Information on data validation was added.
  - *Line 203-207 :“Descriptive analysis was used to identify missing or inconsistent data. Variables with inconsistent scaled responses were recoded to ensure they were consistently scaled. A correlation matrix was constructed to identify multicollinearity. Prior to combining the variables, such as the N95 and KN95 into high protection and cloth and surgical masks into low protection, both a dimensional reduction and reliability analysis was run.”*

**2. Please compact abstract and conclusion part.**

- The abstract is already short at only 167 words. We checked other published papers, and most of the abstracts exceeded our word count. Can the reviewer please clarify?
- The conclusion was rewritten to reduce the word count and streamline the information.
  - *Line 373-379: “ Understanding why masks that offer lower protection are chosen is important in the development of effective exposure prevention measures during a pandemic. The authors illustrated that factors such as sex, comfort, trust, and perceived effectiveness of the mask or respirator can significantly impact the choice of high protection offered by respirators or the lower protection of masks. The knowledge gained will aid in creating targeted communications and outreach aimed at building trust in vulnerable populations, to increase the effectiveness of exposure control measures, such as the choice of higher protection offered by respirators.”*
